# Development of a novel anti-tuberculosis nanodelivery formulation using magnesium layered hydroxide as the nanocarrier and pyrazinamide as a model drug

**DOI:** 10.1038/s41598-022-15953-6

**Published:** 2022-08-18

**Authors:** Bullo Saifullah, Palanisamy Arulselvan, Sharida Fakurazi, Thomas J. Webster, Naeemullah Bullo, Mohd Zobir Hussein, Mohamed E. El Zowalaty

**Affiliations:** 1 Nanomaterials Synthesis and Characterization Laboratory, Institute of Nanoscience and Nanotechnology, Universiti Putra Malaysia, Serdang, 43400 Selangor Malaysia; 2 Department of Human and Rehabilitation Sciences, The Begum Nusrat Bhutto Women University Sukkur, Sindh, 65170 Pakistan; 3 Laboratory of Vaccine and Immunotherapeutics, Institute of Bioscience, Universiti Putra Malaysia, Serdang, 43400 Selangor Malaysia; 4 Department of Human Anatomy, Faculty of Medicine and Health Science, Universiti Putra Malaysia, Serdang, 43400 Selangor Malaysia; 5 Present Address: School of Health Sciences and Biomedical Engineering, Hebei University of Technology, Tianjin, China; 6 Department of Neurology, Jinnah Postgraduate Medical Center Karachi, Sindh, 75510 Pakistan; 7 Present Address: Zoonosis Science Center, Department of Medical Biochemistry and Microbiology, Uppsala University, Uppsala, SE 75 123 Sweden

**Keywords:** Biomaterials, Nanobiotechnology, Biomaterials, Drug delivery

## Abstract

Designing and synthesizing biodegradable drug delivery systems are key research areas in biomedical nanotechnology. Here, we report the development of biodegradable magnesium-layered hydroxide (MgLH) based nanodelivery systems using magnesium oxide (MgO) as the precursor by a precipitation method. The designed nanocarrier does not contain any trivalent metal ions, which are most commonly used for the synthesis of layered double hydroxides (LDHs). The designed delivery system was characterized in detail using X-ray diffraction (XRD), Fourier transform infrared (FTIR) spectroscopy, Thermogravimetric analysis (TGA), Transmission electron microscopy (TEM) and inductively coupled plasma (ICP) analyses. The anti-tuberculosis (anti-TB) drug pyrazinamide (PZA) was successfully intercalated into interlayer galleries of MgLH, resulting in the formation of the nanocomposite, PZA-MgLH, having an average size of about 107 ± 24 nm with a uniform circular shape. The in vitro release of PZA in a human body simulated phosphate buffer saline (PBS) solution was sustained (i.e., almost 66 h) and followed a pseudo-secondorder kinetic model. Moreover, the designed nanodelivery system was found to be highly biocompatible with human normal lung cells (MRC-5) and with 3T3 fibroblast cells as controls for 24 and 48 h. Lastly, the PZA-MgLH nanocomposite showed good anti-tuberculosis activity against *Mycobacterium tuberculosis* and both the PZA-MgLH nanocomposite and its released free drug PZA showed antibacterial activity against tested Gram-positive and Gram-negative bacteria with percentage inhibition ranging from 5.6% to 68% against *S. aureus, E. coli*, and *P. aeruginosa* for the PZA free drug, and 32% to 32.5% against *E. coli* for the PZA-MgLH nanocomposite. In summary, the present results provide significant evidence that the designed nanodelivery system can be used for the delivery of PZA and, thus, should be investigated further for a wide range of anti-TB applications.

## Introduction

We consume magnesium (Mg^+2^) every day in the form of green vegetables, since it is the central metal ion present in the chlorophyll of vegetables and plants. Magnesium is a vital divalent mineral for humans that activates more than 300 enzyme systems in particular for energy metabolism. Magnesium is an important component of soft tissue and bones, and it is the most abundant divalent cation in human cells^1–3^. Despite such promise, few drug delivery vehicles have taken advantage of magnesium in medicine.

The present study formulated magnesium into layered double hydroxides (LDHs) for novel drug delivery applications. The basic structure of LDHs is similar to hydrotalcite by replacing some of the divalent cations with trivalent cations, such as Al^+3^, Fe^+3^, Cr^+3^, etc., to create a positive charge in the inorganic layers^4–6^. This positive charge is balanced by counter anions between the layers, such as nitrate, carbonates, etc., and water molecules. In addition, anions, such as organic drugs or any negatively charged ions, can also be intercalated in these inorganic interlayers^7^. Layered hydroxide salts (LHS) have a similar chemical structure to LDHs, except that there are only divalent metal cations used without any trivalent cations. LHS based on zinc brucite-like layers can be easily prepared using zinc nitrate salts or zinc oxide^8–10^. Magnesium layered hydroxide (MgLH) has a layered structure in which the hydroxide ions are surrounded by the magnesium ion in an octahedral manner. Infinite layers are formed by these octahedral units by edge-sharing with hydroxide ions at right angles to the planes of the layers. In a three-dimensional view, layers stack on top of one another^11^.

Due to the above attractive properties for the use of magnesium in medicine, MgLH was investigated in the present study as a novel drug delivery vehicle for tuberculosis (TB). TB is a potentially serious infectious disease caused by *Mycobacterium tuberculosis*^12^. It has existed for centuries and current technologies still have not been able to eradicate it^13,14^. Tuberculosis remains a worldwide public health threat and it is estimated that 10 million people are infected with TB every year as compared to around 1.2 million HIV-negative people who died in 2020^15^. There have been no new drugs developed for TB for over five decades and the currently best available anti-TB drug treatment lasts from 6 to 24 months, depending on the type of TB. Treating TB with drugs is complicated by the adverse effects associated with anti-TB drugs, multiple drug regimens and the long treatment necessary^16^. These three factors cause significant patient non-compliance to the drugs developed for TB, which is the most common cause of treatment failure^16,17^. Clearly, we need effective drug formulations to treat TB that can sustain drug release over a long period of time making such drugs easier to achieve patient compliance.

Pyrazine 2-carboxamide commonly known as pyrazinamide (PZA), is a first-line anti-TB drug with patients required to take about 500 mg of PZA daily^18^. Although it is one of the most powerful anti-TB drugs, its administration has been limited to certain lower doses as it has many reported side effects such as liver injury, anorexia, arthralgia, malaise, vomiting, dysuria, urticaria, pruritis and skin rashes. PZA is not recommended for the treatment of latent TB due to its high rate of liver toxicity^18^. Biocompatible and biodegradable drug-delivery systems with sustained-drug release properties can be very useful in improving drug bioavailability^19,20^. The improved bioavailability of the drugs would not only result in better therapeutic efficacy but would also reduce dosing frequency and thereby would improve patient compliance, avoiding one of the major causes of treatment failure for TB^16,21,22^.

In this in vitro study, we developed magnesium layered hydroxides as a drug delivery system without the addition of any trivalent ions in the inorganic layers. To the best of our knowledge, the synthesis of MgLH drug nanodelivery systems by a co-precipitation method using magnesium oxide as the precursor has not been previously reported. Using this newly developed method, we successfully intercalated the anti-TB drug, PZA. The non-toxic cellular characteristics of MgLH and its degradation product, Mg^**+2**^, make it a strong candidate for various biomedical applications, such as drug delivery, gene delivery, biosensing and bioimaging, for TB and numerous other diseases^16^.

## Materials and methods

### Chemicals

Analytical-grade chemicals were used without any further purification. Magnesium oxide (MgO) and methanol were purchased from Sigma-Aldrich (Saint Louis, MO, USA) and Ajax Finechem (Sydney, Australia), respectively. Deionized water was used in all of the experimental work.

### Method of preparation

Magnesium oxide (0.5 g) was added to a 50 mL mixture of methanol and water (40 mL methanol + 10 mL water) containing 1 g of PZA. The mixture was stirred for 15 min under a nitrogen flux. A sodium hydroxide solution (0.2 M) was added dropwise until the pH became basic and a white precipitate was formed. The white precipitate was washed thoroughly with water and subsequently with methanol, after which the sample was subjected to drying at 60 °C for one day. In the end, the sample was ground to a powder and subjected to further characterization as described below.

### Characterization

A Shimadzu XRD-6000 diffractometer (Tokyo, Japan) was used for X-ray diffraction (XRD) studies. CuK_α_ radiation at 30 kV and 30 mA was used to record the Powder X-ray diffraction (PXRD) patterns in the 2θ range of 2–60°. Fourier transform infrared (FTIR) spectra of the materials were recorded over a range of 400–4000 cm^−1^ on a Perkin-Elmer 100 series spectrophotometer by a direct sample method (Perkin-Elmer, Waltham, MA, USA). For thermogravimetric and differential thermogravimetric analyses (TGA/DTG), a Mettler Toledo instrument (Greifensee, Switzerland), was used. For the thermal analysis, samples were subjected to heating from 25 to 1000 °C with a temperature increase rate of 10°C per minute, under a nitrogen environment. The morphology of the sample surface was studied by a field emission scanning electron microscope (FESEM), JOEL JSM-6400 (Tokyo, Japan). A Shimadzu UV-1650-PC UV/Vis spectrophotometer was used to determine the optical properties. The UV/VIS spectroscopy and controlled-release studies were done under atmospheric conditions. For the loading quantification of PZA in MgLH, a Sykam HPLC system was used with an auto-injector, Sykam 5300, a Sykam S3250 UV/VIS detector and a Sykam quaternary pump system 5300 (Sykam Gmbh, Eresing, Germany), with a Zorbax Rx-Sil column of 4.6 × 150 mm and with a 5 μm particle size (Agilent, CA, USA). The dynamic light scattering (DLS) technique was applied using a Zeta sizer nanoseries, NANO-S Malvern instrument, for the determination of the nanocomposite particle size distribution.

### Sustained-release analysis

Human body simulated 0.1 M phosphate buffer saline (PBS) solutions at pH values of 7.4 (blood pH) and 4.8 (intracellular lysosomal pH) were used to study the sustained release of PZA from the inorganic galleries of MgLH. Approximately 0.4 mg of the nanocomposites was placed into 3.5 mL of pH 7.4 and 4.8 PBS solutions, and the absorbance at a wavelength *λ*_max_ = 270 nm was selected on the UV/VIS spectrophotometer to study the release of PZA from the nanocomposites.

### Cell culture

Human lung fibroblast MRC-5 (ATCC® CCL-171™) and 3T3-Swiss albino (ATCC® CCL-92™) cells were purchased from the American Type Culture Collection (ATCC) (Manassas, VA, USA), and the cells were cultured in Dulbecco’s modified Eagle medium and RPMI 1640 media containing 10% fetal bovine serum. The growth media contained 100 units/mL penicillin and 50 µg/mL streptomycin, respectively. The cells were maintained at 37 °C in a humidified atmosphere in the presence of 5% CO_2_.

### Antimicrobial susceptibility test

Drug susceptibility testing of the nanocomposites was carried out as previously reported using the BBL non-radiometric fluorescence-based method of MGIT 960 against *Mycobacterium tuberculosis* (ATCC® 25618™), and the MICs of the nanocomposites were determined as previously described^23,24^. The Mycobacteria Growth Indicator Tube (MGIT) with BACTEC MGIT 960 growth supplement for drug susceptibility testing was used in an MGIT 960 instrument (Becton Dickinson Diagnostic Systems, Sparks, MD, USA) as described previously^23–25^.

### Microbial growth inhibition kinetics

The antimicrobial effect and activity of the as-synthesized nanocomposite PZA-MgLH on the growth kinetics of Gram-positive bacteria *Staphylococcus aureus* (ATCC®43300™)*,* Gram-negative bacteria *Pseudomonas aeruginosa* (ATCC®27853™)*,* and *Escherichia coli* (ATCC®25922™)*,* and yeast *Candida albicans* (ATCC®20408™) (ATCC, Manassas, VA, USA) were determined as previously described^23,26–28^. The percentage inhibition of each nanocomposite against each microorganism was calculated according to the following equation as previously described:

1 $${\text{Inhibition rate }} = { 1 } - \, \left[ {{\text{CFU}}_{{{\text{treated}}}} /{\text{OD}}_{{{\text{control}}}} } \right] \times {1}00$$

The efficiency of the nanocomposites to inhibit the growth of the microorganisms was determined by the differences in the equivalent number of colony-forming units before and after treatment as the percentage of microbes that were inhibited by the nanocomposites; this was calculated from the previous equation^23,26–28^.

### Statistical analysis

The experimental biological assays were conducted from three different independent investigations and expressed as the mean ± standard deviation (SD). Data were presented as the mean ± SD and P < 0.05 and P < 0.001 were considered statistically significant. Significant differences were examined using analysis of variance (ANOVA) with SPSS 20.0 software (SPSS Inc., Chicago, IL, USA). GraphPad Prism statistical software version 6.01 (GraphPad, San Diego, CA, USA) was used for data management and statistical analysis.

## Results and discussion

### XRD and spatial orientation of the drug

**Figure 1:**
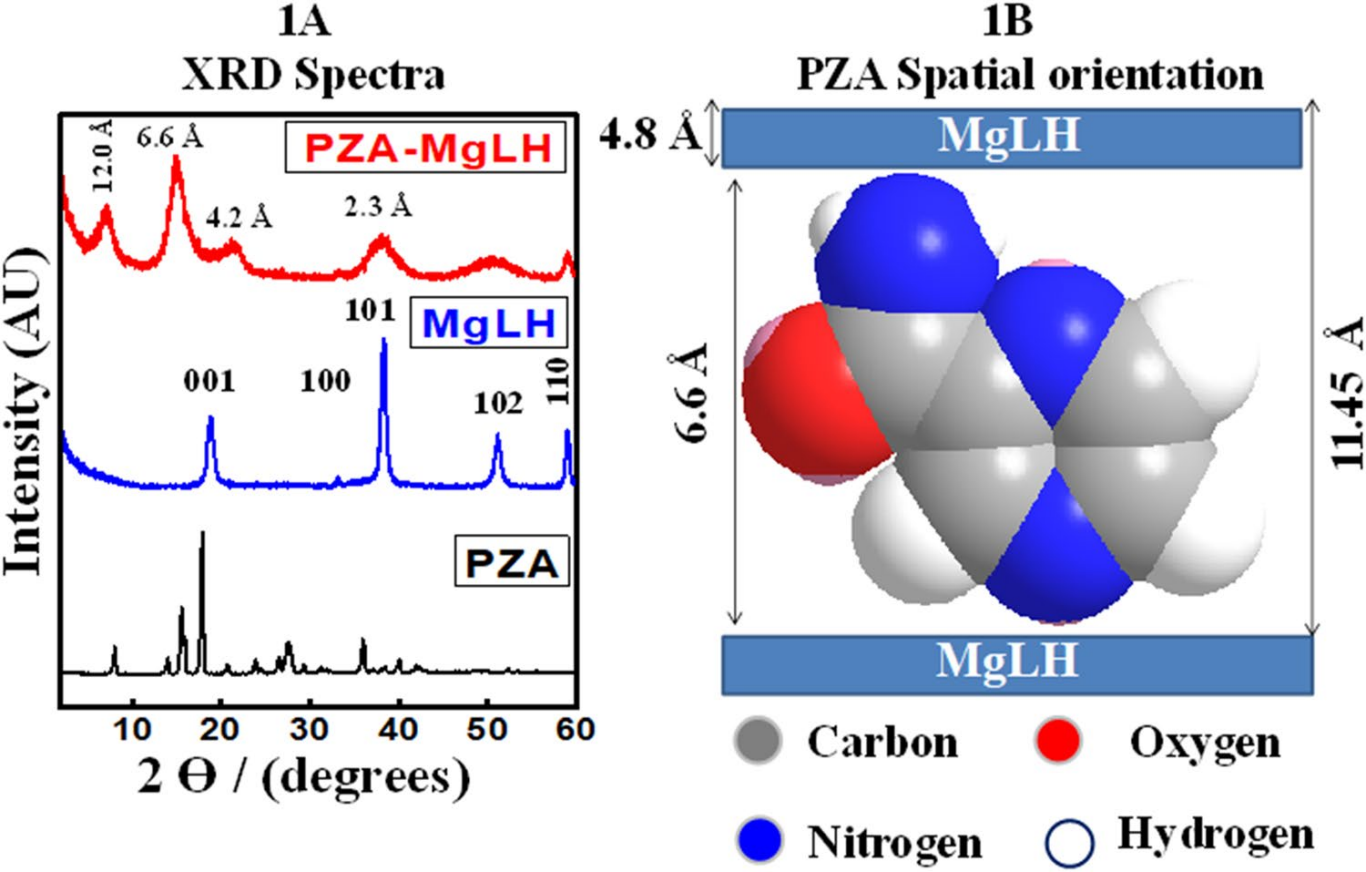
(**A**) XRD pattern of PZA, MgLH and PZA-MgLH nanocomposite and (**B**) the spatial orientation of PZA between the MgLH inorganic interlayers.

Figure 1 shows the XRD spectra of the free drug, pyrazinamide (PZA), magnesium layered hydroxides (MgLH) and a PZA-MgLH nanocomposite. MgLH showed a typical XRD pattern with characteristic reflections of 001, 100, 101 and 102, at 2ϴ values of about 19°, 33°, 38° and 52°, respectively^16,29^. It is well known that the basal spacing of MgLH is 4.8 Å. In the PZA-MgLH nanocomposite, the basal spacing increased from 4.8 to 12.0 Å, whereas the increase in the basal spacing was strong evidence of the successful intercalation of PZA into the interlayer galleries of MgLH. The average basal spacing of PZA-MgLH was found to be 11.45 Å from the XRD results. The crystallinity for the MgLH before PZA loading was determined to be 98% and for the nanocomposite PZA-MgLH, it decreased to 92% indicating a successfully fabricated nanocomposite. The 3D molecular size of PZA was determined using Chem draw software; the vertical, horizontal and thickness of PZA were found to be 6.6 Å, 8.1 Å and 2.9 Å, respectively. The spatial orientation of PZA in the interlayer galleries was determined by subtracting the layered thickness of MgLH, i.e. 4.8 Å, from the average basal spacing of the PZA-MgLH nanocomposite which was 11.45 Å. After subtracting the layered thickness of MgLH, the remaining value obtained was 6.6 Å which is equal to the vertical size of PZA. This suggests that PZA is vertically oriented in the interlayer gallery of the MgLH. The vertical orientation is the most feasible form, from an electrostatic interaction point of view as there will be more chances of interaction between PZA and the MgLH inorganic layers. Figure 1B schematically shows the orientation of PZA into the MgLH interlayer gallery.

### Fourier transform infrared (FTIR) analysis

**Figure 2:**
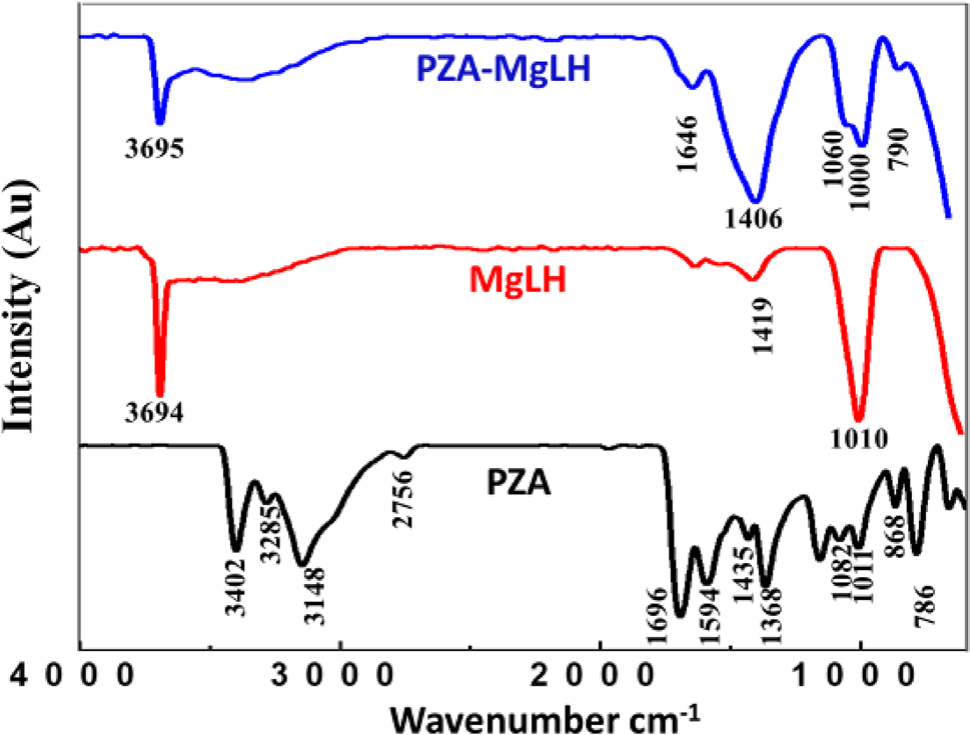
FTIR spectra of free pyrazinamide (PZA), magnesium layered hydroxides (MgLH) and the PZA-MgLH nanocomposite. AU = arbitrary units.

Figure 2 shows the Fourier Transform Infrared (FTIR) spectra of the free drug PZA, the carrier, the MgLH and the nanocomposite, PZA-MgLH. The FTIR spectrum of the free drug, PZA, showed characteristic functional group vibration bands, such as asymmetric and symmetric bands of the terminal amine group, N–H at 3402 cm^−1^ and 3285 cm^−1^, respectively. The carbonyl (C=O) stretching band of the characteristic amide group of PZA is observed at 1696 cm^−1^. The other characteristics of the PZA bands, such as C–N, C–C, C–H and C–N–C bands, are also present^30,31^. The FTIR spectrum of MgLH shows the characteristic vibrational bands of the hydroxyl (O–H) group at 3694 cm^−1^, and another broadband is observed at 3000–3400 cm^−1^ which is due to the strongly hydrogen-bonded water molecules. The MgOH deformation band is also observed at 1010 cm^−1^^16,29,32^. In the FTIR spectrum of the nanocomposite PZA-MgLH, vibrational bands for both PZA and MgLH are present with slight shifts in their position. The presence of the characteristic functional group bands of PZA and MgLH in the PZA-MgLH nanocomposite complimented the XRD results, indicating the successful intercalation of PZA into the interlayer galleries of MgLH. The details of the vibration bands of free PZA, MgLH and PZA-MgLH are given in Table 1.

**Table 1:** The FTIR absorption bands of PZA, MgLH and PZA-MgLH.

Assignment	PZA	MgLH	PZA-MgLH
Hydroxyl group of MgLH	–	3694^32^	3695^32^
Mg–OH stretching	–	3000–3400^32,33^	Overlapped Mg–OH stretching
N–H assym: stretching	3402	–	
N–H sym: stretching	3285	–	
C–H stretching	3148	–	
C–H (wag)	2756	–	
C=O stretching	1696	–	1664
C–C (ring) stretching	1594	–	–
C–N (ring) assym: stretching/Mg–OH bending	1435^30,31^	1419^32,33^	1406
C–N (ring) sym: stretching	1368	–	–
C–C stretching	1082	–	1060
C–N stretching/Mg–OH deformation bending	1011	1010^33^	1000
C–N–C bending	868	–	–
C–N bending	786	–	790

### Elemental analysis and drug loading

The XRD and FTIR results were further complemented by ICP-OES and HPLC analyses. The percentage of Mg in the composites was determined using inductively coupled plasma (ICP-OES) and the amount of PZA loading was determined by high-performance liquid chromatography (HPLC) analyses. Based on the ICP-OES results, 58% of Mg was found to be present in the nanocomposite, PZA-MgLH.

### HPLC analysis

The percentage loading of the active drug, PZA, was determined using the HPLC technique, as was previously reported with slight modifications^34^. In brief, the following parameters were used; the oven temperature was set at 30 °C and the mobile phase was set at a flow rate of 1 mL/min. The retention time of PZA was found to be 2.2 min and a wavelength of 235 nm was selected for the UV detector. The mobile phase was acetonitrile (A) and a potassium dihydrogen phosphate buffer at 15 mmol L^−1^ with the pH adjusted to 4.0 ± 0.1 with phosphoric acid (B). The samples were run in an isocratic mobile phase comprising a ratio of 89:11 for the A: B solvents. The standard drug, PZA solutions, and the samples were prepared in 5 mL of a 1 molar hydrochloric acid solution and 45 mL of the mobile phase. The calibration curve was obtained by running the standard solutions of PZA (i.e. 0.0, 20, 40, 60, and 80 mg/mL) and the R^2^ was found to be 0.99. The percent loading of PZA was found to be 22.42%.

### Thermogravimetric analysis

**Figure 3:**
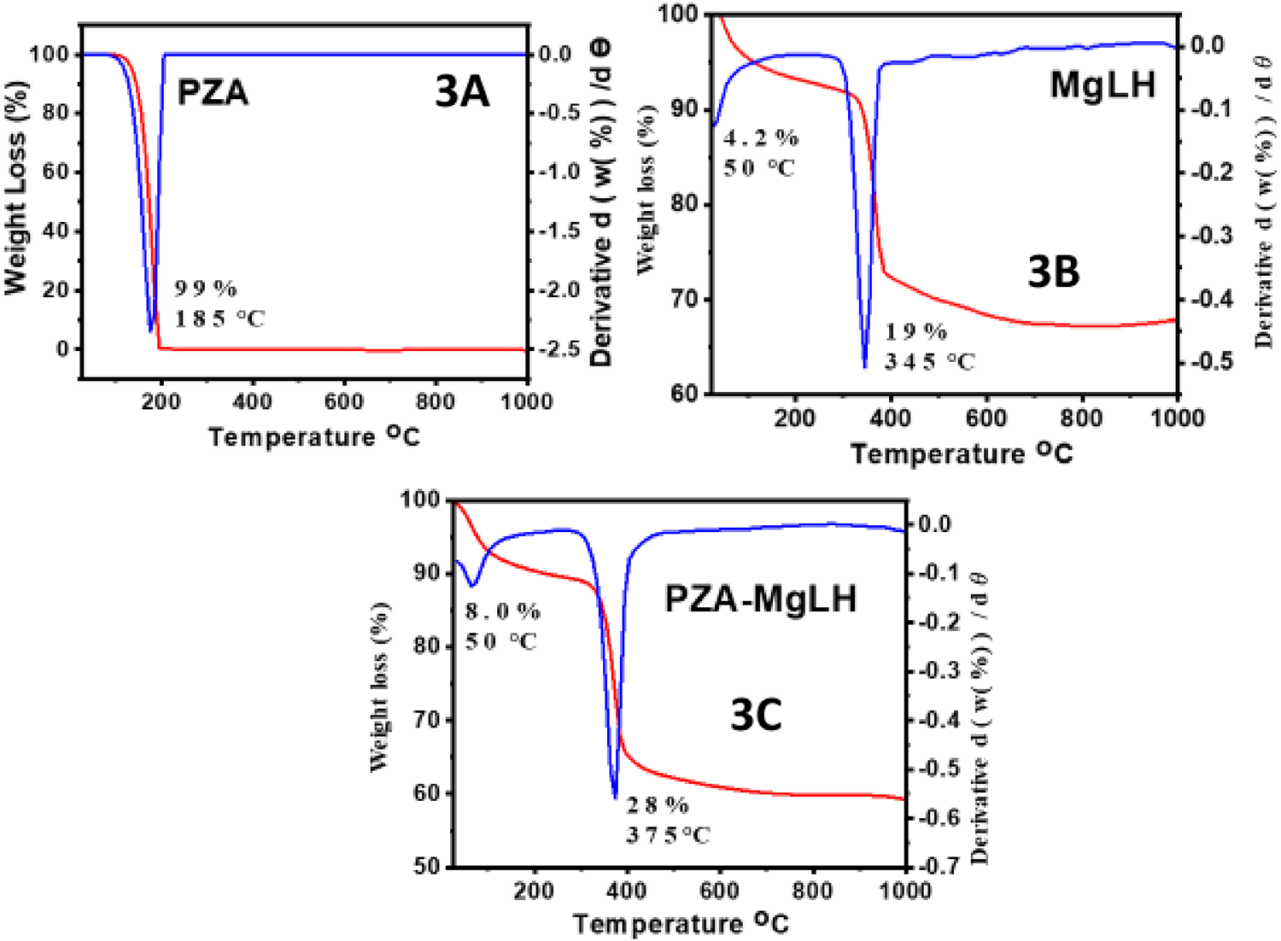
TGA/DTG thermograms of the free drug, PZA (**A**), MgLH (**B**) and PZA-MgLH (**C**).

The thermal stability of PZA, MgLH and PZA-MgLH was examined using the thermogravimetric analysis and differential thermogravimetric analysis (TGA/DTG). Figure 3A–C shows the thermograms of the PZA, MgLH and PZA-MgLH nanocomposites, respectively. The free drug, PZA, was decomposed at 185 °C with a weight loss of 99%, as shown in Fig. 3A. Figure 3B shows the TGA/DTG analysis of the MgLH empty carrier, with two weight loss events, the first one occurred at 50 °C with a weight loss of 4.2%, which can be ascribed to physically adsorbed water. The second event occurred at 345 °C with a weight loss of 19%, due to the dehydroxylation of MgLH as shown in Fig. 3B. For the PZA-MgLH nanocomposite, two weight loss events were observed, the first one occurred at 50 °C with a weight loss of 8.0%, due to the loss of physically adsorbed water. The second thermal event of weight loss started from 270 to 450 °C with a major event at 375 °C with a weight loss of 28%, which can be ascribed to the thermal decomposition of PZA and dehydroxylation of MgLH as shown in Fig. 3C. The thermal stability of PZA was enhanced from 185 °C to 270 °C when it was intercalated in between the MgLH inorganic brucite-like layers of the nanocomposite, PZA-MgLH.

### In vitro release and kinetics

**Figure 4:**
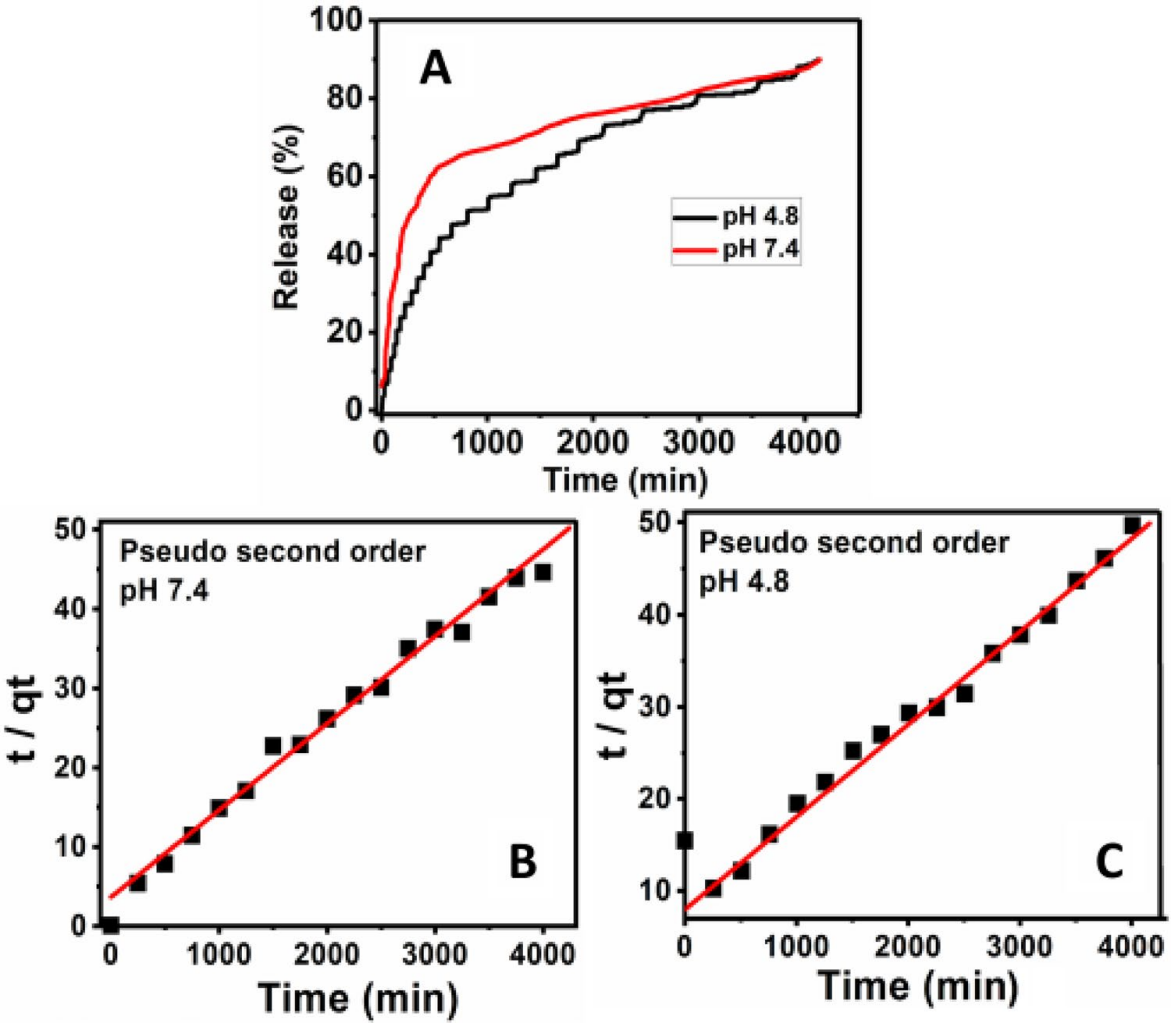
In vitro release of PZA and its kinetic fitting for the pseudo-second-order model; (**A**) the in vitro release of PZA from PZA-MgLH nanocomposites in PBS solutions of pH 7.4 and pH 4.8, kinetic fitting of PZA in vitro release in pseudo-second-order model at pH 7.4 (**B**) and 4.8 (**C**).

The in vitro release of PZA from the PZA-MgLH nanocomposites was investigated in a human body simulated phosphate buffer saline (PBS) solution of pH 7.4 and pH 4.8 under a constant temperature of 37 °C. Under both physiological conditions of pH 7.4 and pH 4.8, the drug PZA took about 4000 min for complete release as shown in Fig. 4A. The overall percentage release of PZA in PBS solutions of pH 7.4 and pH 4.8 was found to be 90%, and the release pattern was similar under both conditions. The drug release behavior from MgLH was found to be similar to the drug release from layered double hydroxides (LDHs) and zinc layered hydroxides^16,35–37^. The overall release of PZA from MgLH was highly sustained under both conditions of PBS pH 7.4 and pH 4.8.

For the kinetic study of PZA release, three different kinetic models namely a pseudo-first-order, pseudo-second-order and parabolic diffusions were used, which are described in detail in the literature^35–37^. The in vitro release of PZA from the PZA-MgLH nanocomposite in both PBS solutions of pH 7.4 and pH 4.8 followed a pseudo-second-order, with a correlation coefficient (R^2^ value) of 0.99. Figure 4B,C represents the kinetic fitting of the PZA in vitro release for a pseudo-second-order release and Table 2 lists the correlation coefficient (R^2^) for all the three kinetic models used and the rate constant obtained for the pseudo-second order.

**Table 2:** Correlation coefficient (R^2^) and rate constants (k) obtained by fitting data of the release kinetics of PZA from PZA-MgLH into PBS solutions at pH 4.8 and 7.4.

Samples	pH	% Release	R^2^			Pseudo-second order
			Pseudo-first-order	Pseudo-second-order	Parabolic diffusion model	Rate constant, K (mg/min)
PZA-MgLH	7.4	90	0.54	0.99	0.76	2.05 × 10^–5^
PZA-MgLH	4.8	90	0.69	0.99	0.67	2.25 × 10^–5^

### Transmission electron microscopy of the PZA-MgLH nanocomposites

**Figure 5:**
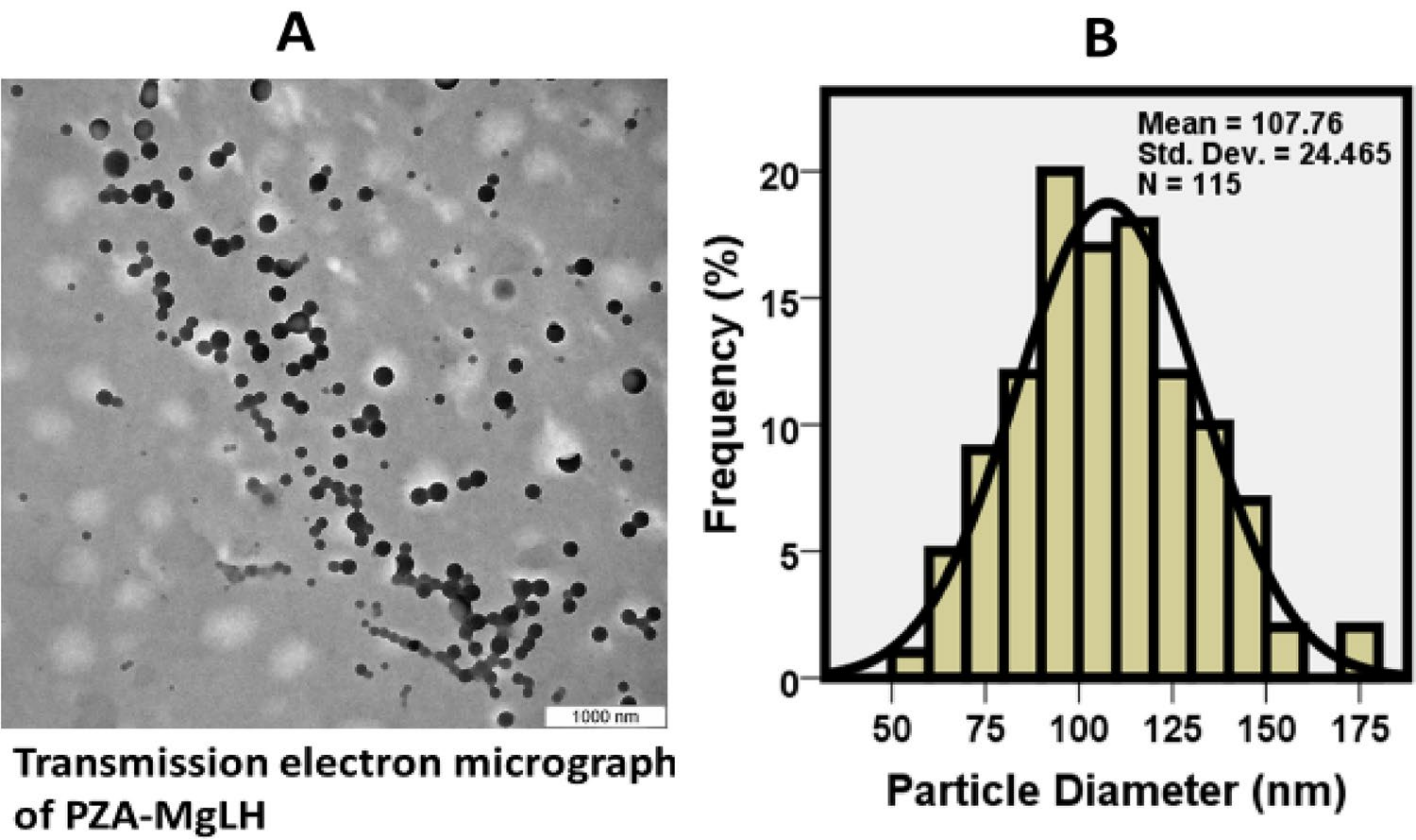
(**A**) Transmission electron micrograph of PZA-MgLH with a scale bar of 1000 nm and (**B**) particle size distribution of around 107 nm for PZA-MgLH obtained using ImageJ software.

The particle size of the nanocomposite, PZA-MgLH, was determined from TEM images and its distribution was determined using Image-J. About 115 particles (N) were randomly selected from the TEM images and the diameter was measured. It can be observed that the particles were well dispersed and uniform in round shapes (Fig. 5A), with an average diameter size of about 107 ± 24 nm (Fig. 5B).

### Anti-mycobacterial and antimicrobial assays

**Figure 6:**
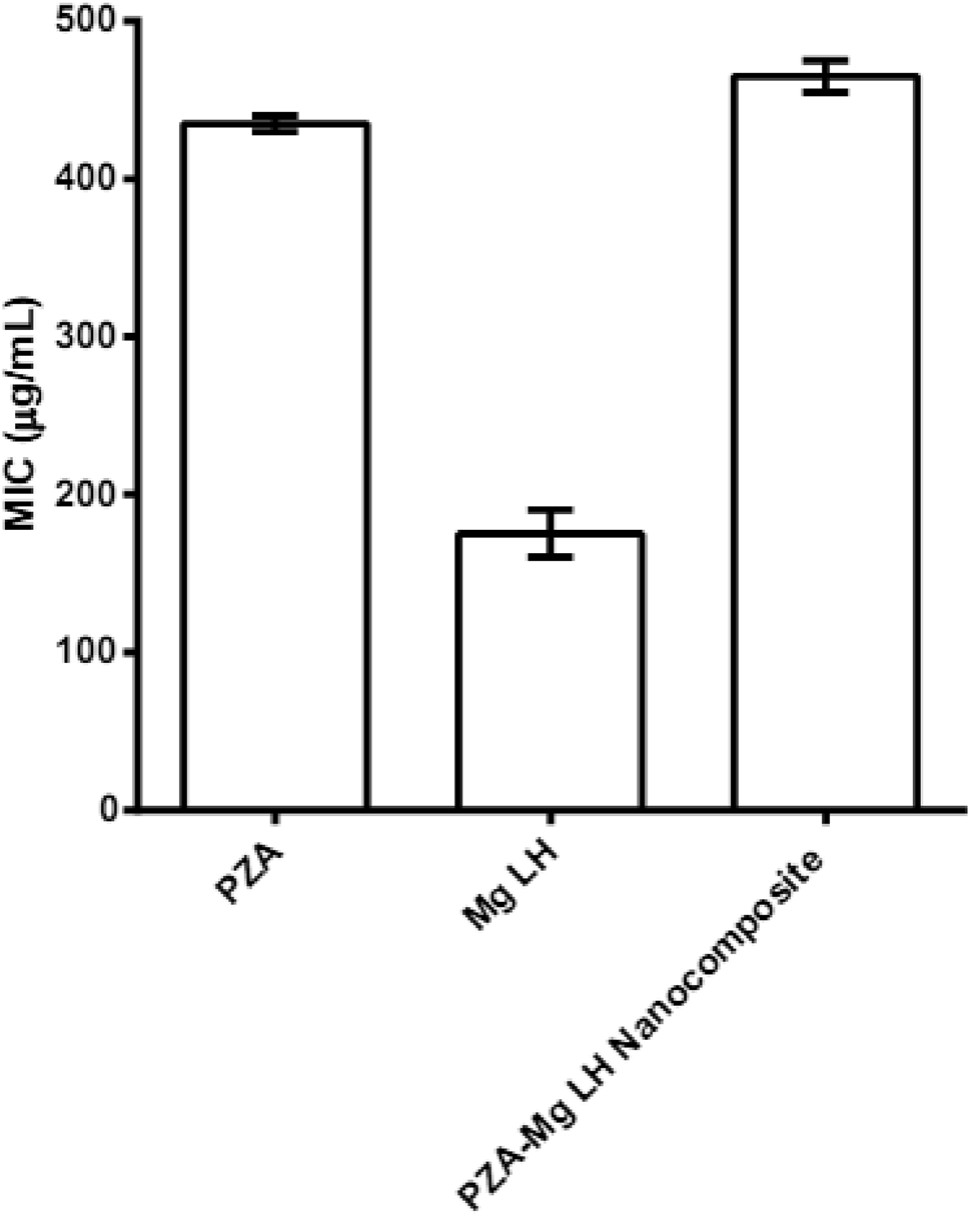
Minimum inhibitory concentrations (MICs) (µg/mL) of Mg-LH and PZA-MgLH nanocomposites as compared to the free drug PZA against *Mycobacterium tuberculosis* as determined using the Mycobacteria Growth Indicator Tube (MGIT) with BACTEC MGIT 960 growth supplement for drug susceptibility testing and measured using the MGIT 960 instrument (Becton Dickinson Diagnostic Systems, Sparks, MD, USA). Data = mean ± SEM; N = 3; all values statistically (p < 0.01) different than each other.

Using the highly efficient and accurate system of BACTEC MGIT 960, the MICs of the as-synthesized PZA-MgLH nanocomposite against *Mycobacterium tuberculosis* was determined as shown in Fig. 6. The MICs were found to be 465 µg/mL compared to 435 µg/mL for the free drug (PZA). Although the MIC of the nanocomposite was slightly higher compared to the free drug, the actual amount of PZA present was 465 µg/mL which was much smaller based on percent loading. The actual amount of PZA present in a 465 µg/mL nanocomposite was 102 µg/mL, determined based on the percentage of drug loading. The effective therapeutic concentration (MIC) of PZA in PZA-MgLH (i.e.,102 µg/mL) was almost 4 times lower than the free drug, PZA. This improved efficacy can be attributed to the nanoscale size of the PZA-MgLH nanocomposite and its sustained release properties.

### Antimicrobial analysis

**Figure 7:**
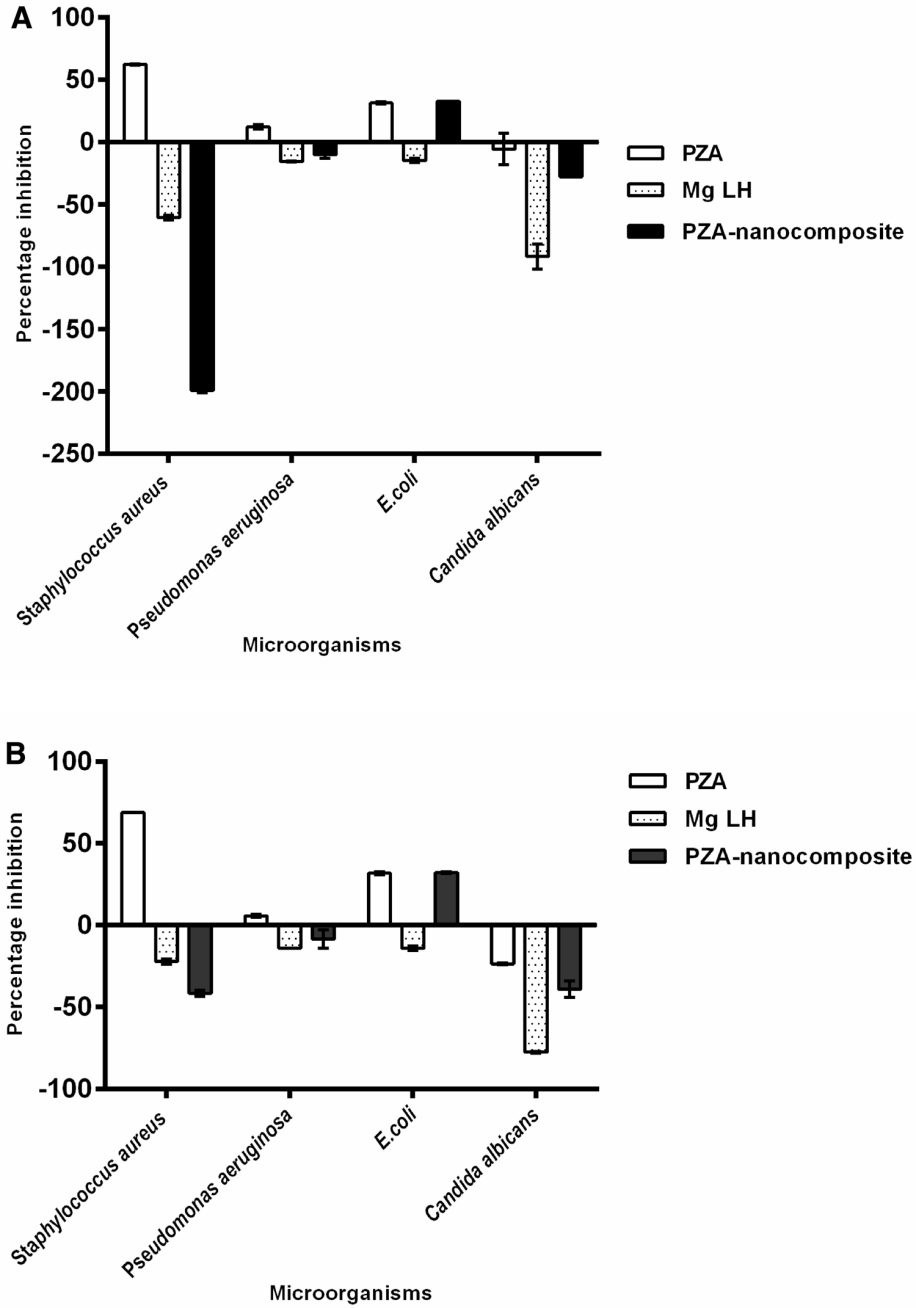
Effects of PZA-MgLH nanocomposites and the free drug PZA on the microbial growth of Gram-positive (*S. aureus*), Gram-negative (*P. aeruginosa* and *E. coli*) bacteria, and *Candida albicans* using the plate colony-forming unit (CFU) counting method at two different concentrations: (**A**) 1 mg/ml and (**B**) 2 mg/ml. Data = mean ± SEM; N = 3; all values statistically (*p* < 0.01) different than each other for each respective bacteria.

The results of the antimicrobial testing showed that the PZA-MgLH nanocomposite and the free drug, PZA, have antibacterial activity against Gram-positive bacteria and Gram-negative bacteria, but lack activity against *Candida albicans*, as shown in Fig. 7 (A and B), as indicated from the percentage inhibition of each compound against the different tested microorganisms, with a percentage inhibition ranging from 5.6 to 68% against *S. aureus, E. coli*, and *P. aeruginosa* for the PZA free drug, and 32 to 32.5% against *E. coli* for the PZA-MgLH nanocomposite, at both concentrations tested. This additional antibacterial activity of the PZA-MgLH nanocomposite and its release of PZA free drug is advantageous in the treatment of polymicrobial infections that might be associated with a TB infection.

### Cytotoxicity test

**Figure 8:**
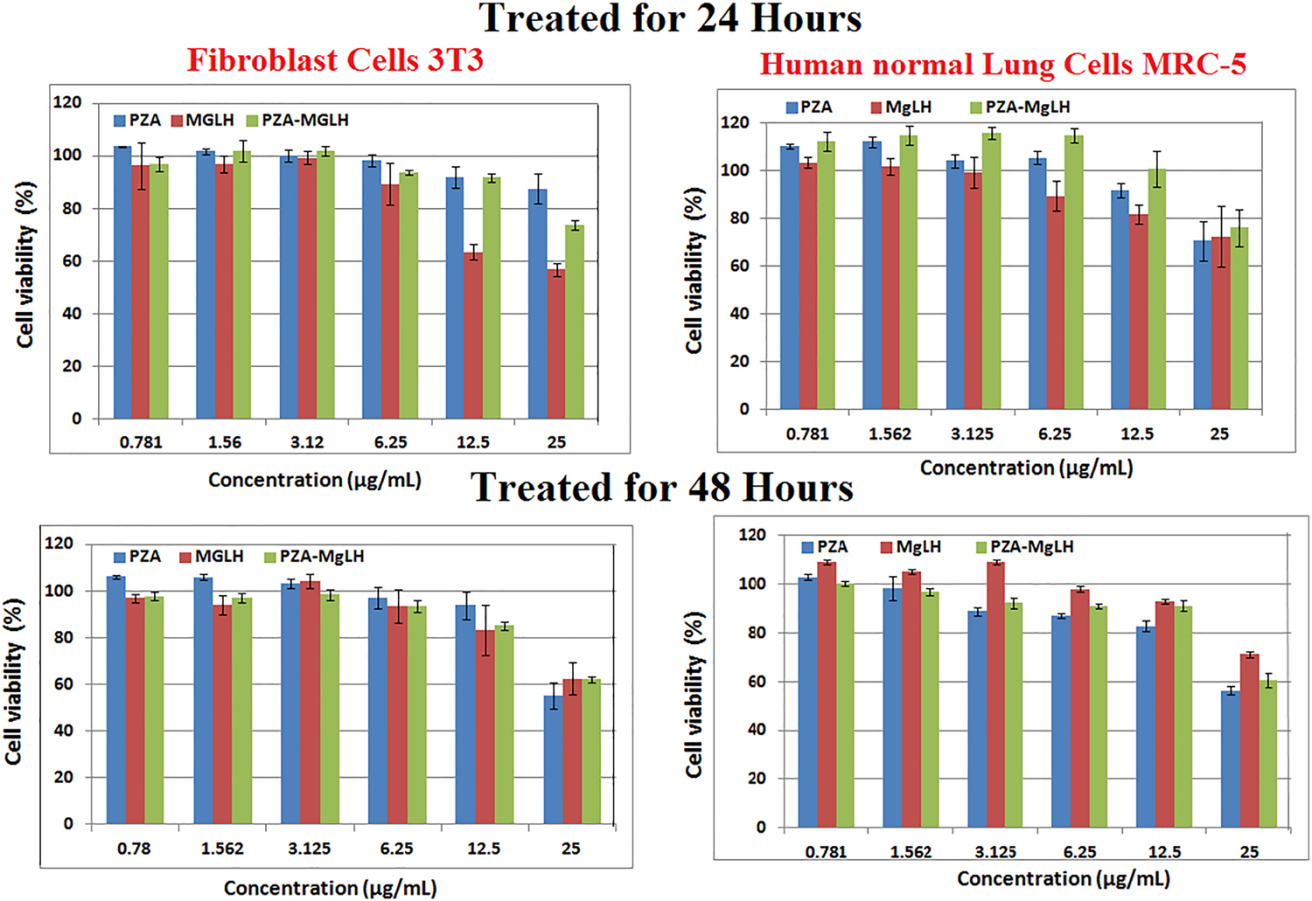
Viability of 3T3 fibroblast cells and MRC-5 human normal lung cells after 24 and 48 h of incubation. Data = mean ± SEM; N = 3; all values are statistically (p < 0.01) similar within each material formulation at each concentration, except for the MgLH at 12.5 μg/mL and higher against fibroblast cells.

Lastly, to ensure the non-toxic nature of the free drug and its nanocomposite formulation, cytotoxicity assays were conducted. The cytotoxicity of the free drug PZA, the carrier MgLH and the nanocomposite PZA-MgLH was determined by incubating them with human normal lung cells MRC-5 and 3T3 fibroblast cells, according to the previously reported cytotoxicity assay protocol^35–37^. Different concentrations of the samples ranging from 0.78 to 25 μg/mL were incubated with the above-mentioned cell lines for different periods of 24 h and 48 h. All of the samples were found cytocompatible with both types of normal cells, however, a slight cytotoxic effect was observed at a higher concentration of 25 μg/mL for the carrier MgLH which can possibly be due to the nitrate counter anion present in MgLH, but even at this concentration, the cell viability was found to be about 60% for the carrier MgLH. Thus, we conclude that all the samples including free PZA, the nanocarrier, MgLH and the nanocomposite, as well as PZA-MgLH have nearly similar cytocompatibility with human normal cells MRC-5 and fibroblast cells 3T3 (Fig. 8).

## Conclusion

Here, we report for the first time, the development of novel magnesium layered hydroxides using MgO as the precursor and for the synthesis of a drug nanodelivery system for the anti-tuberculosis drug, pyrazinamide (PZA). The in vitro release of PZA from its MgLH host was found to be sustained in human body simulated solutions. The designed PZA-MgLH nanocomposite showed better anti-TB activity and was also found to possess antimicrobial activities against Gram-positive and Gram-negative bacteria. The nanocomposite, PZA-MgLH, the host, MgLH, and the guest, PZA, were found to be biocompatible with human normal lung cells and 3T3 fibroblast cells. Magnesium is one of the most important elements that are present in the human body, and magnesium based nanomaterials open up a new horizon for low toxicity, highly efficient drug delivery systems in the field of biomedical nanotechnology, especially TB as noted here. Further, pre-clinical experiments will be needed to confirm its anti-TB potential without undesirable side effects.

## Data Availability

The datasets used and/or analyzed during the current study are available from the corresponding author upon reasonable request.
